# Application of Metabolomic Tools for Studying Low Molecular-Weight Fraction of Animal Venoms and Poisons

**DOI:** 10.3390/toxins10080306

**Published:** 2018-07-24

**Authors:** Agnieszka Klupczynska, Magdalena Pawlak, Zenon J. Kokot, Jan Matysiak

**Affiliations:** Department of Inorganic & Analytical Chemistry, Poznan University of Medical Sciences, Grunwaldzka 6 Street, 60-780 Poznan, Poland; magdalepaw@gmail.com (M.P.); zkokot@ump.edu.pl (Z.J.K.); jmatysiak@ump.edu.pl (J.M.)

**Keywords:** venom, poison, metabolites, mass spectrometry, nuclear magnetic resonance, bufadienolides

## Abstract

Both venoms and poisonous secretions are complex mixtures that assist in defense, predation, communication, and competition in the animal world. They consist of variable bioactive molecules, such as proteins, peptides, salts and also metabolites. Metabolomics opens up new perspectives for the study of venoms and poisons as it gives an opportunity to investigate their previously unexplored low molecular-weight components. The aim of this article is to summarize the available literature where metabolomic technologies were used for examining the composition of animal venoms and poisons. The paper discusses only the low molecular-weight components of venoms and poisons collected from snakes, spiders, scorpions, toads, frogs, and ants. An overview is given of the analytical strategies used in the analysis of the metabolic content of the samples. We paid special attention to the classes of compounds identified in various venoms and poisons and potential applications of the small molecules (especially bufadienolides) discovered. The issues that should be more effectively addressed in the studies of animal venoms and poisons include challenges related to sample collection and preparation, species-related chemical diversity of compounds building the metabolome and a need of an online database that would enhance identification of small molecule components of these secretions.

## 1. Introduction

Animals have developed many mechanisms to adapt to their role as victim or predator. One of these adaptations is venom production in specialized glands or cells and its injection into the target animal via a wound. In addition to venomous animals, there are also poisonous species that deliver their toxins in a passive way (their body contains the poisonous substance). Both venoms and poisonous secretions are complex mixtures that assist in defense, predation, communication and competition in the animal world [1,2]. They consist of variable bioactive molecules, such as proteins, peptides, salts and also metabolites, e.g., amines, amino acids and alkaloids [3]. A venom or poison from a single animal may contain hundreds of different components with a wide range of biological functions [4].

Venomous and poisonous animals have been the focus of human interest for years, due to the danger associated with them and curiosity how such inconspicuous animals can be so powerful in their activity [3]. The exceptional properties of venoms and poisons depend on their composition, which is species specific. Initially, before the introduction of mass spectrometry techniques and bioinformatics, studies of poisonous and venomous secretions were very tedious, required a large number of samples, huge sample volume and time-consuming methodologies. They were also limited by the sensitivity of the methods used. Only major proteins could be successfully identified and knowledge of minor proteins and other compounds was insufficient [5].

Studies on animal venoms and poisons have been continuously enhanced by technological progress opening up opportunities to discover new bioactive molecules. Introduction of “omics” technologies for venom research, such as proteomics, transcriptomics, genomics and metabolomics, enabled large­scale data collection and analysis. In analogy to the above-listed technologies, the new term “venomics” was coined. Venomics can be defined as the study of animal venom and venom glands by using highly sensitive and high-throughput techniques [1]. In the beginning, the word “venomics” referred only to proteomic analyses of venoms [6,7]. In 2006 a project on studying full genomes, transcriptomes and proteomes from several venomous animals was described with this term [8]. Later on “venomics” was also used to describe smaller-scale studies that combined proteomics and transcriptomics [2,5,9,10].

Metabolomics, the latest of the “omic” technologies, aims at identifying and quantifying the entire set of metabolites present in cells, biological fluids and tissues (called metabolome). Endogenous metabolites are at the end of a series of processes beginning with the genome followed by the transcriptome and proteome. Therefore, metabolome fills the gap between a given genotype and phenotype. Metabolome components include such compound classes as amines, amino acids, organic acids, steroids, alkaloids, and sugars. The term “metabolomics” was introduced in 1999 by Nicholson, Lindon, and Holmes [11]. Since then, technological advances have enabled rapid development of metabolomic research. Analytical techniques used in these studies include nuclear magnetic resonance (NMR) and mass spectrometry (MS) based techniques [12,13].

The importance of metabolomics is still increasing regarding the range and frequency of its application [14]. Metabolomics is currently used to examine venom and poison components (occurring even at extremely low concentrations) as well as to better understand their biological properties. Some reports also present the venom impact on living organisms at the metabolome level [15,16,17,18]. 

## 2. Aims and Methods

The aim of this article is to summarize the available literature where metabolomics was used in studies on venom or poison composition. First, we present an overview of the strategies used for examining the metabolic content of these secretions. Special attention is paid to the classes of metabolites identified in various venoms and poisons and potential applications of the small molecules discovered. Key challenges of metabolomic investigations of these kinds of biological matrices are also discussed.

PubMed was searched to identify works with a “metabolomics” tag or those concerning metabolic profiling, mainly using chromatography, NMR and MS-based methodologies. Additional papers were found in the references of those works. This review encompasses research focused on poisons and venoms, both fresh or processed, collected from toads [19,20,21,22,23,24], frogs [25,26], spiders [27,28,29,30,31], snakes [32,33], and scorpions [23] ants [34,35,36] and wasps [37]. The studies included were divided into two the most commonly used metabolomic strategies: untargeted and targeted ones (Table 1). Articles presenting metabolome profiling and the effects of venom or selected venom components on living organisms or cell lines were not considered, as we were interested only in venom composition studies. Data on other venom and poison components (peptides, proteins) and their activities are not presented as they are beyond the scope of the article. 

## 3. Metabolomics Strategies Used for the Analysis of Poisons and Venoms

**Figure 1:**
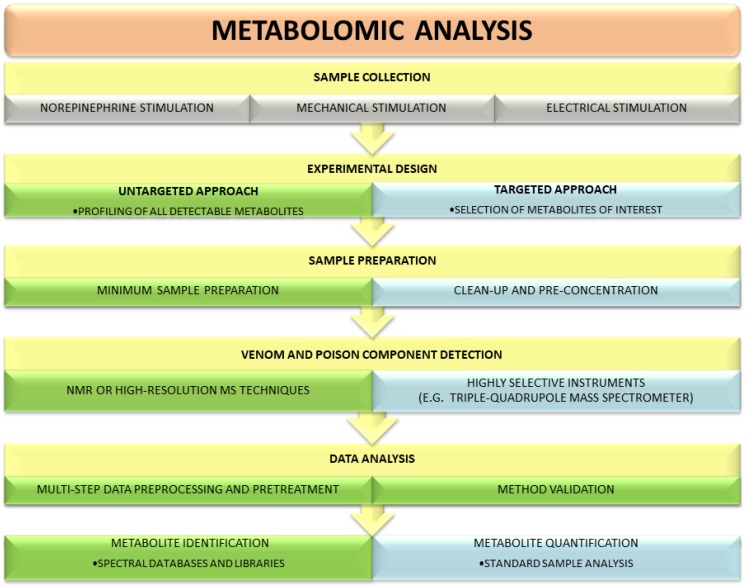
Comparison of untargeted and targeted metabolomic workflows used in studies on venoms and poisons. The main differences include sample preparation method, data preprocessing and processing, and the level of metabolite quantification.

There are two main strategies used in metabolomic research: untargeted and targeted (Figure 1). These two approaches provide complementary information on specimens and employ different analytical platforms. Untargeted metabolomics relies mainly on NMR or high-resolution MS techniques, whereas targeted experiments require highly selective instruments, such as a triple-quadrupole mass spectrometer [38]. Each strategy has its inherent advantages and disadvantages. The goal of an untargeted experiment is to globally profile all detectable metabolites in a specimen. Compared with a targeted analysis, it requires neither sophisticated sample preparation procedures nor knowledge of the metabolites that are in the analyzed sample. Due to its comprehensive nature, the untargeted approach generates large datasets and requires extensive data preprocessing and pretreatment as well as advanced chemometric techniques (multivariate statistics) for data analysis. After receiving a unique, complete metabolic profile of a studied venom or poison, identification of individual signals is usually carried out using different databases, spectral libraries and available standards. Targeted metabolomics focuses on the isolation and quantification of a defined group of metabolites and thus utilizes dedicated methodologies [39,40]. It offers greater selectivity and sensitivity than untargeted strategy. Unambiguous identification of the investigated molecules limits the risk of artifacts during analytical and statistical experiments [39,41]. 

Given the characteristics of both strategies, the untargeted one is usually applied first for an analysis of an unknown sample, as it offers the opportunity of studying the whole metabolic content of the sample and detection of unanticipated molecules. By employing untargeted methodologies signals from various compounds, such as amino acids, amines, and lipids, are registered; however, quantitative data are not acquired. A targeted approach is used for answering a specific biological question and is usually limited to one class of metabolites. In a selection of compounds of interest, knowledge of the sample components is needed.

### 3.1. Untargeted Metabolomic Studies

Schroeder et al. [27] applied untargeted metabolomics for an analysis of spider venoms. Their study involved venoms of more than 70 species from 27 families of spiders. Venoms were acquired by electrical stimulation and then lyophilized. Before analysis the samples were dissolved in D_2_O, then the solutions were sonicated and centrifuged. The supernatant was used for NMR analysis. After NMR experiments, selected samples were prepared for high-performance liquid chromatography coupled with mass spectrometry (HPLC-MS) analysis by lyophilization, redissolution in water and filtration. The combination of several analytical techniques identified a broad range of small molecules in the spider venom samples that included amino acids (glutamic acid), polyamines (spermidine), organic acids (citric acid, isocitric acid, γ-amino butyric acid), neurotransmitters (acetylcholine, octopamine, histamine) and sulfated nucleosides.

A noteworthy example of the metabolomic assay is the study of Hu et al. [23], who analyzed secretions from a living scorpion and a living toad. They used field-induced direct ionization mass spectrometry for real-time detection of metabolites released from those animals. The scorpion’s venom was collected from a living animal by mechanical stimulation of its tail. The raw secretion was directly introduced to the MS inlet. The MS spectra identified lysine, serotonin and tryptophan, and some lipids. Toad’s glands were stimulated with a needle to receive a milky gland secretion. The secretion was transferred within a methanol-filled capillary to the MS inlet. The approach identified such small molecules as serotonin, *N*-methylserotonin and bufadienolides, e.g., bufotenin, bufotenindine, gamabufotalin, bufotalidin, and cinobufotalin.

The untargeted strategy was also used for toad poison analysis by Gao et al. [21]. They compared the composition of secretions of different toad species and different origin: commercial toad poisons prepared from *Bufo bufo gargarizans* and *Bufo melanosticus* and fresh toad poisons obtained from *Bufo viridis*, *Bufo bufo gargarizans*, *Bufo melanosticus*, and *Bufo marinus*. A total of 17 samples were extracted with methanol using ultrasound at room temperature. After centrifugation, the poisons were analyzed by HPLC and HPLC coupled with triple quadrupole mass spectrometry. Twenty bufadienolides were identified by comparison of the retention times, ultraviolet (UV) spectra and mass spectra with those of reference substances. Further 23 compounds were determined by interpretation of the MS/MS fragmentation patterns. The substances identified included suberoyl arginine, free bufadienolides, sulfated bufadienolides and suberoyl esters of bufadienolides. All samples of commercial toad poisons contained a similar number and type of compounds. The major components were six bufadienolides: gamabufotalin, arenobufagin, telocinobufagin, bufotalin, cinobufotalin and resibufogenin. All 10 batches of commercial poison were very similar to a fresh sample obtained from *Bufo bufo gargarizans*. The poisons from Indonesian and Malaysian *Bufo melanosticus* differed in the type of bufadienolides. The poison analysis of *Bufo marinus* enabled identification of only four bufadienolides and showed no differences between female and male individuals. It was, therefore, concluded that the metabolite content in the poison does not depend on sex. The largest variety of bufadienolides was detected in *Bufo viridis* poison and only three compounds (telocinobufagin, marinobufagin, bufalin) were common in all analyzed samples. The chemical diversity of the poisons examined could be due to species-specific differences, application of various sample preparation processes e.g., drying, and response to environmental factors, such as habitat.

Ma et al. [19] investigated changes in *Bufo bufo gargarizans* glandular secretion induced by vacuum-drying at 60 °C and air-drying at room temperature. Fresh gland secretion was obtained by stimulation. The experiment involved three groups of poison samples: vacuum-dried at 60 °C for 12 h, air-dried at room temperature for 24 h and fresh skin secretion. All samples were extracted with methanol by ultrasonication prior to metabolite profiling with ultra-high performance liquid chromatography-quadrupole time-of-flight mass spectrometry (UHPLC-Q-TOF-MS). Principal component analysis (PCA) and orthogonal projection to latent structures discrimination analysis (OPLS-DA) were used to visualize differences and similarities among the groups. In the fresh and dried poison, 31 identified metabolites belonged to bufadienolides. Vacuum-drying at 60 °C gently changed the levels of bufadienolides vs. fresh poison, whereas air-dried poison experienced a dramatic loss of bufadienolides. The quality of air-dried poison compared with vacuum-dried one at 60 °C was worse mainly in terms of conjugated bufadienolide content. The drop in bufadienolide content may also be caused by enzymes capable of degrading these compounds. They reduced bufadienolide levels during air-drying at room temperature but were deactivated during vacuum-drying at 60 °C. Furthermore, the study showed how the drying method affected the anti-tumor activity of the toad poison in human hepatocellular carcinoma SMMC-7721. The vacuum-dried skin secretion was a more potent inhibitor of cancer cell proliferation than the skin secretion air-dried at room temperature [19].

Another example of untargeted metabolomic analysis of *Bufo* genus involves eight poisons from subgenus *Rhinella* (*R. jimi*, *R. crucifer*, *R. marina*, *R. schneideri*, *R. icterica*, *R. granulosa*, *R. major*, and *R. margaritifera*) and one from subgenus *Rhaebo* (*R. guttatus*) [22]. Secretions obtained by manual compression of the macroglands were extracted with 0.1% trifluoroacetic acid in water, sonicated and centrifuged. HPLC with UV or MS detection was used to identify the components of glandular secretions. The compounds in *Rhinella* and *Rhaebo* poisons were of variable abundance and they are listed in Table 2. Apart from obvious species-related differences, the secretions of toads from completely different habitats had similar HPLC profiles. Moreover, the secretions from related species sharing the same habitat were significantly different in terms of alkaloid and steroid content. These differences did not correlate with diet, morphology (size of the body and parotid glands) or habitat.

Cavalcante et al. [25] performed a metabolomic assay of the skin secretion of another poisonous amphibian, *Dermatonotus muelleri*. The substance was obtained by electrical stimulation and diluted in ultrapure water. The next steps involved lyophilization and storage at −20 °C until analysis and filtration. The metabolomic profiling was comprised HPLC, MS and NMR. The HPLC profile of *D. muelleri* skin secretion indicated mainly peaks of polar compounds, however, most of them were not identified. In MS analysis tryptophan was identified as the major constituent. ^1^H NMR spectrum yielded chemical shifts typical for sugars. The applied methodology identified neither alkaloids nor steroids in that poisonous secretion. The authors also tested biological activity of the identified low molecular weight compounds. None of them inhibited bacterial growth and in fact, the fraction containing tryptophan promoted microbial propagation.

The untargeted strategy was also employed to investigate the skin secretions of frogs belonging to *Pipidae* family. Mariano et al. [26] characterized the previously unexplored composition of *Pipa carvalhoi* skin secretions and compared the secretions from bondage and wildlife frogs, and with and without norepinephrine stimulation. The first batch of samples was collected via norepinephrine stimulation of the glands, the second via mechanical stimulation, and the third was harvested without any stimulation. All samples were lyophilized, suspended in 5% acetonitrile containing 0.1% trifluoroacetic acid and filtered just before HPLC, MS and NMR analysis. The major low-molecular weight component in *P. carvalhoi* skin secretion was kynurenic acid, a metabolite with neuromodulatory activity and multiple targets in the brain and periphery [42]. Regardless of the animal origin, diet and stimulation method, the chromatographic profiles were the same except for the peak intensity that indicates quantitative but not qualitative differences.

An interesting group of low molecular-weight compounds identified in spider venoms is acylpolyamines. A great number of acylpolyamines have been discovered in different spider genera and their chemical structures were elucidated using NMR and MS techniques [28,29,30,31]. The typical structure of acylpolyamines contains an aromatic moiety at one end, and either a guanidine group or a primary amino group at the other [43].

Piperidine alkaloids are characteristic components of venoms of fire ants in the genus *Solenopsis*. Studies of venom alkaloid chemistry of *Solenopsis* species were performed using an untargeted tool—gas chromatography-mass spectrometry (GC-MS) [34,35,36]. Chen and Fadamiro [35] performed re-investigation of *S. richteri* venom composition and demonstrated the presence of both *cis* and *trans* alkaloids. In addition to the previously described components, they identified seven novel alkaloids, including three Δ^1,2^-piperideines and four Δ^1,6^-piperideines. Lai et al. [34] studied the effects of temperature and season on the composition of *S. geminata* venom and indicated that the ratio of *cis* C_11_ to *trans* C_11_ isomers in the venom of minor workers was higher in spring compared to winter.

### 3.2. Targeted Metabolomic Studies

Aird et al. [32] applied a targeted approach to analyze polyamine content (spermine, spermidine, putrescine and cadaverine) in snake venom. The samples originated from three families of snakes: *Elapidae*, *Viperinae*, *Cortalinae* (in total 31 venoms were analyzed). Polyamines were derivatized with 4% benzoyl chloride and quantified using UHPLC-TOF-MS. Compound identity was confirmed based on compliance of retention time and mass-to-charge ration with those registered for standards. The presence of polyamines was determined in all samples but at significantly different levels. Elapid venoms contained fewer amines than other venoms: spermine was almost completely absent and no spermidine was found, whereas putrescine was the most abundant. Despite large variations in polyamine levels between viperid and cortalid venoms, spermine and spermidine occurred at the highest amount in these taxa.

Torres et al. [37] determined free amino acids in venom of the social wasp *Polistes lanio*. Venom reservoirs were collected from three hundred females of *P. lanio* and macerated in ultrapure water to expel the venom. Fresh and frozen venom samples were analyzed using the HPLC method. Before HPLC analysis, amino acids were derivatized by treatment with diethyl ethoxymethylenemalonate (DEEM). The investigation allowed for identification of 10 amino acids and quantification of 8 of them. Among the studied analytes, an abundance of arginine, alanine, threonine and serine was observed in wasp venom.

The targeted strategy was also used for determination of bufadienolides, which are promising anticancer components of toad poison [44,45]. Meng et al. [20] examined the content of bufadienolides in *Bufo bufo gargarizans* secretions using UHPLC-TOF-MS. Samples were prepared by liquid–liquid extraction and divided into two fractions: one with bufogenin and one with bufotoxins. The study identified bufogenins as dominant components in toad gland secretion, with bufotalin, bufalin, cinobufagin and resibufogenin as the most abundant compounds. The prevailing bufotoxins included bufalin-3-*O*-succinate-arginine, bufalin-3-*O*-pimelate-arginine and bufalitoxin. The authors also confirmed cytotoxic effects of the toad poison extracts and demonstrated strong cytotoxicity of bufogenins (higher than bufotoxins) in tumor cell lines.

Drying is a technique commonly used for the protection and storage of venom and poison samples. However, the drying method may affect sample quality. Therefore, Zhou at al. [24] employed targeted HPLC-MS/MS and pattern recognition approach to assess the effects of different methods on the levels of 36 bufadienolides in glandular secretions of *Bufo bufo gargarizans*. They tested four drying variants: vacuum-drying at 60 °C, freeze drying, air-drying at 60 °C, and air-drying at room temperature. The vacuum-drying at 60 °C yielded a sample of the highest quality regarding the content of free and conjugated bufadienolides. The poison specimen obtained after freeze-drying was also of high quality. Contrary to that, air-drying at room temperature caused a dramatic loss in both types of bufadienolides. These data correlate with a report by Ma et al. [19].

Snake bufadienolides were also a subject of scientific scrutiny. Hutchinson et al. [33] analyzed a secretion of defensive glands (nuchal gland fluid) located on the skin of the Asian snake *Rhabdophis tigrinus*. They obtained the secretion from two groups of snakes: living on toad-rich and toad-free Japanese islands. The methanolic solutions of the nuchal gland fluid were analyzed for qualitative and quantitative evaluation using the NMR and HPLC methods, respectively. The gland secretion from the snakes inhabiting a toad-rich island contained bufadienolides due to the presence of toads (rich in bufadienolides) in their diet. By contrast, the gland secretion of the snakes from the toad-free island did not contain bufadienolides. The authors concluded that *R. tigrinus* is unable to synthesize bufadienolides and can only acquire these steroids from the consumed toads.

## 4. Biological Significance and Potential Applications of Small Molecule Components of Poisons and Venoms

**Figure 2:**
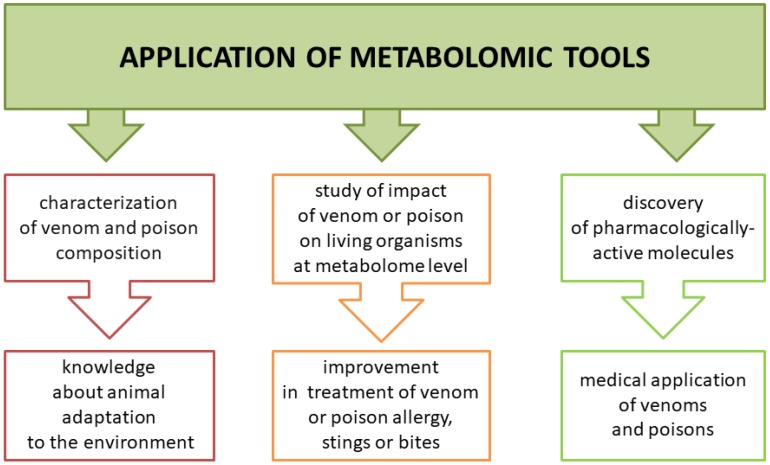
Three major areas in venom and poison research that might benefit from the application of metabolomic technologies.

Animal poisons and venoms are complex mixtures of biologically active compounds that can serve as a weapon and/or protection. Metabolomic analysis of those secretions can help us understand how animals adapt to the environment but also how they affect their prey, including humans. This knowledge may be highly useful in improving treatment in case of a sting or bite by venomous animals or contact with poisonous species (Figure 2). Another benefit is the possibility of using venom or poisonous secretion as a source of pharmacologically active molecules. Low molecular components of venoms and poisons have a variety of functions. Some studies showed their cytotoxic activity against tumor cells, making them potential anti-cancer drugs. However, some studies lacked a control group of normal cell lines to confirm selective cytotoxicity of venoms or poisons against cancer cells [19,20].

Analyses of low molecular-weight fractions of venoms and poisons from different animals indicated that some common components and also specific molecules occur in those secretions. Among unique components are bufadienolides in toad poisons, acylpolyamines in spider venoms and piperidine alkaloids in fire ant venoms, whereas free amino acids and monoamines are present in many types of venomous and poisonous secretions. Each component has its own role in the immobilization of a prey. However, it should be noted that constituents of venoms and poisons can work synergistically and the biological activity of single components can vary in comparison to a whole secretion.

Polyamines are present in many venoms, including those of snakes and spiders. Their concentrations differ depending on the metabolic pathway they originate from. For example, cadaverine is produced in an anabolic pathway that is separated from the pathway that yields the other three polyamines (spermine, spermidine, putrescine). Most venoms probably do not trigger a systemic response in the prey due to too low polyamine level but local effects are possible. Spermine has numerous pharmacological functions including promotion of hypotension, introduction of negative inotropic in the cardiac muscle, interaction with receptors: calcium release receptors, nicotinic acetylcholine receptors, muscarinic receptors, GABAA receptors, sensitization of acid-sensing ion channels and capsaicin receptors, inhibition of Ca^2+^-ATPase resulting in skeletal muscle paralysis and cell death, and promotion of the blood-brain barrier breakdown. Spermidine, putrescine, and cadaverine greatly affect the biological activity of histamine, which explains their hypotensive effect. The polyamine mode of action in the prey organism, which includes hypotension, circulatory shock and direct paralysis, allows the snakes or spiders to hunt their prey easier [32,46]. Polyamines may be applied in anticancer therapy in the future. Wilson et al. [47] showed that polyamines from tarantula venom exhibit selective toxicity against MCF-7 breast cancer cells (other tested cell lines were SK-MEL-28 (melanoma) and human neonatal foreskin fibroblasts (NFF). The aromatic head group of polyamines plays an important role in this activity.

Acylpolyamines are major low molecular-weight components of spider venoms. These compounds are targeted on ionotropic glutamate receptors and, therefore, affect neuron communication and block neuromuscular junctions. Additionally, acylpolyamines inhibit polyamine transport and take part in preventing the toxic effect of an overabundance of polyamines in cells. The potential medical applications of acylpolyamines isolated from spider venom are associated with their neuroprotective action against ischemia and influence on spinal receptors which regulate hyperalgesic states [48].

Amino acids present in the secretions of spiders, scorpions, toads and frogs, can serve as precursors of monoamines. Both classes of metabolites have variable biological functions in venoms or poisons. Lysine and tryptophan, found in the venom of the scorpion *Tityus serrulatus*, contribute to prey immobilization and prevent its escape [49]. Glutamic acid is a neuroactive compound that may paralyze the prey. This effect was observed in insects, where due to their small size and structure of their circulatory system glutamic acid acts directly and rapidly. In mammals it is quickly metabolized in the liver [50]. Octopamine, a monoamine detected in spider venoms, is a neurotransmitter and contributes to prey paralysis by modulating muscle dynamics and overactivating the sympathetic nervous system. The second function of monoamines is inflicting pain or discomfort to deter a predator and in defense. This effect may be provided by histamine and serotonin. Another function of monoamines is facilitating the spread of venom components in the victim’s body: octopamine increases heart rate, whereas serotonin and histamine cause vasodilation [51].

Bufadienolides belong to the best-known poison components. They are present not only in the secretions of amphibians, such as *Bufo*, *Rhinella* and *Rhaebo* toads but also in Asian snake venom [33] and arthropods [52]. Bufadienolides also occur in the plant kingdom [53,54]. They are steroids with a characteristic α-pyrone ring at C-17 position. Depending on the substituted forms at C-3, there are two groups of bufadienolides: bufogenin (unconjugated steroid) and bufotoxin (steroid conjugates), e.g., suberoyl arginine esters [52,55]. Toads use poison as a last resort, not to attack but only to defend. It is not deadly for people but it may be cardiotoxic and cause heart dysfunction and arrhythmias [56]. Bufadienolides may also exert antiangiogenic, hypertensive or anti-hypertensive, immunosuppressive, anti-endometriotic and positive ionotropic effects [57]. Some studies showed antiparasitic [58], antiviral [59] and antimicrobial activity of those molecules [60]. Given this broad spectrum of biological activity, toad poison of different species is the major component of some popular traditional Chinese medicines, e.g., ChanSu [52]. The most interesting trait seems a promising antitumor activity of bufadienolides including inhibition of cell proliferation and migration, induction of cell differentiation, inhibition of cancer angiogenesis, induction of apoptosis, disruption of the cell cycle, reversal of multi-drug resistance and enhancement of cytotoxic drug activity. One of the proposed molecular mechanisms of this anticancer activity is the inhibition of Na^+^/K^+^-ATPase [61,62,63]. However, medical use of bufadienolides is highly restricted due to their narrow therapeutic margins, short drug half-life and toxicity [64].

## 5. Challenges and Future Perspectives

Metabolomics opens up new perspectives for the study of venoms and poisons as it gives an opportunity to investigate their previously unexplored low molecular-weight components. Advances in high-throughput “omic” methodologies (especially mass spectrometry techniques) contribute greatly to the characterization of venom and poison composition. Despite using state-of-the-art analytical technologies, metabolomic experiments encounter numerous difficulties and challenges.

Untargeted metabolomics allows for analysis of a wide range of small molecules but it also struggles with many challenges. The major bottleneck in non-targeted metabolomic studies is the reliable identification of detected signals. The problem of annotating the detected molecular also occurs as specific compounds occur in many metabolomic studies of animal venoms and poisons. In an analysis of low molecular mass fraction (<10 kDa) of *Dermatonotus muelleri* skin secretion, only one constituent was successfully identified as tryptophan because the remaining molecules yielded uninformative fragmentation spectra [25]. A similar situation was reported for <10 kDa skin secretion composition of *Pipacar valhoi*—kynurenic acid (tryptophan derivative) was identified as a sole metabolic component of that secretion [26]. Although a mass spectrum obtained by direct ionization mass spectrometry (DI-MS) analysis of a secretion released from a sting of a living scorpion contained many peaks of high abundances, only lysine, tryptophan and serotonin were identified (based on their weight and literature search) [23]. Application of large in-house spectral libraries could increase the number of metabolites identified in analyzed specimens; however, it requires time and financial resources. The cost of standard substances is usually considerable and, furthermore, some of them are not available and have to be synthesized. Moreover, a large volume and complexity of metabolomic data calls for specialized statistical tools. The use of multiple analytical methodologies and necessity of extensive data pre-processing also require advanced bioinformatic solutions.

Metabolites are molecules with a molecular weight below 1500 Da and different physicochemical properties. Metabolites contain classes of compounds with extremely different properties in terms of polarity, pKa, pH, solubility, chemical and thermal stability, etc. [65]. As a result, qualitative and quantitative characterization of animal venoms and poisons at metabolic level presents a great analytical challenge. The method allowing for an analysis of the entire metabolome does not exist. Authors of the studies mentioned in this review used several different techniques to gain more information on the investigated specimens [25,26,27,33]. Each method yielded a different kind of data. The observed differences, i.a. in low molecular-weight fractions of toad secretions, may be due to both biological and analytical reasons (various methodologies applied) [19,21,22]. Only a combination of various platforms allows for gaining wider knowledge on venom and poison composition. Moreover, a variety of physicochemical characteristics is a reason for the lack of a universal methodology for sample preparation and detection of metabolites. Therefore, the choice of a proper analytical strategy in metabolomic research depends on the purpose of the study and class of metabolites selected for analysis. The most commonly used analytical platform in venom and poison metabolomic analyses is liquid chromatography-tandem mass spectrometry (LC-MS) (Table 1). The application of both LC-MS and NMR techniques allows for identification of unknown sample constituents, however MS ensures higher sensitivity than NMR. Therefore, MS allows for broadening the knowledge about venom and poison components, even occurring at very low concentration.

Another challenge concerns the proper handling of specimens. The influence of sample collection and preparation on data obtained in metabolomic experiments was observed in many research studies. Ma et al. [19] and Zhou et al. [24] demonstrated considerable effects of drying method on the quality and quantity of bufadienolides in toad gland secretions. The collection, storage and preparation of poisonous secretions and venom specimens are crucial steps in metabolomic research, especially in quantitative analysis. The first approach to overcome this challenge is real-time monitoring used in the study of scorpion venom and toad poison [23]. Direct introduction of secretions to the MS inlet eliminates the problems with sample preparation. The second approach is using standardized protocols for sample handling, especially in research aimed at the development of new medicines based on venom or poison constituents, antivenom production and desensitization. The application of rigorously standardized procedures will ensure the chemical quality of the products manufactured from venoms and toxins.

A weak point of many investigations presented in this review is a scarce biological interpretation of the results obtained. The articles contain detailed sample preparation protocols, measurement conditions and lists of identified compounds. However, there is no information on biological reasons for the presence of particular metabolites in the venoms or poisons studied. A discussion of the biological relevance of the identified molecules and interpretation of their possible functions should be an important part of each metabolomic report.

Future directions of venom and poison metabolomics include the development of a dedicated database. A well-designed and freely available electronic database would be an ideal solution for storing and organizing data from all metabolomic investigations of various species. One of the most popular metabolomic databases is the Human Metabolome Database (HMDB), an online database that gathers qualitative and quantitative data about thousands of human metabolites [66]. By linking three kinds of data: chemical, clinical, and biochemical, HMDB facilitates reliable metabolite identification and data interpretation. Creation of an open-access database of venom and poison components, including their physiochemical, biological, pharmacological and toxicological properties, would allow us to collect and disseminate the results of studies conducted by various research teams.

## 6. Concluding Remarks

The introduction of “omic” technologies has revolutionized the study of venoms and poisonous secretions. The vast majority of these studies are focused on protein and peptide profiling and only a few publications investigating low molecular-weight fractions of venoms and poisons are available. Although the metabolomic strategies have already been used to analyze the venoms of spiders, snakes and scorpions, as well as toad and frog poisons, there are very few papers on the metabolite content in other animal venoms collected from i.a. honeybee or wasp. Current venomics utilized mainly proteomic and transcriptomic technologies and little is known about small molecules present in insect venoms [67,68,69,70]. Reports indicating the presence of alkaloids in ant venom [71,72] and lysophosphatidylcholines in giant water bug venom [73,74] can be expanded by the application of sophisticated metabolomic tools. Therefore, it can be concluded that the metabolomics of venoms and poisons is still in its infancy.

Many of the cited studies employ LC-MS-based untargeted metabolomics as this strategy provides a broad overview of the low molecular-weight components of venoms and poisons. Metabolomics can greatly increase our knowledge of venomous and poisonous organisms. Understanding the basis of biological activity of their secretions may improve treatment of bites or stings and help to discover molecules with therapeutic potential. Issues that must be better addressed in venom and poison metabolomics include challenges related to sample collection and preparation, difficulties with the chemical identification of detected features, and the creation of an online database. Future perspectives also require the integration of metabolomic data with data from other “omic” platforms.

## Figures and Tables

**Table 1:** A list of metabolomic studies on animal venoms and poisons including strategy, method and example of metabolites.

Venom/Poison Source	Metabolomic Strategy	Method	Example Metabolites	Reference
Spider	Untargeted	HPLC-MS; NMR	Organic acids, nucleosides, amines, amino acids	Schroeder et al. (2008) [27]
Spider	Untargeted	HPLC-MS; NMR	Acylpolyamines	Tzouros et al. (2004) Tzouros et al. (2013) Hisada et al. (1998) Palma et al. (1998) [28,29,30,31]
Snake	Targeted	HPLC; NMR	Steroids (bufadienolides)	Hutchinson et al. (2013) [33]
Snake	Targeted	UHPLC-MS	Polyamines	Aird et al. (2016) [32]
Scorpion	Untargeted	DI-MS	Amino acids, amines	Hu et al. (2013) [23]
Toad	Untargeted	HPLC; HPLC-MS	Amino acids, steroids (bufadienolides)	Gao et al. (2010) [21]
Toad	Untargeted	DI-MS	Amines, steroids (bufadienolides)	Hu et al. (2013) [23]
Toad	Untargeted	HPLC; HPLC-MS	Alkaloids, steroids (bufadienolides)	Sciani et al. (2013) [22]
Toad	Untargeted	UHPLC-MS	Steroids (bufadienolides)	Ma et al. (2016) [19]
Toad	Targeted	UHPLC-MS	Steroids (bufadienolides)	Zhou et al. (2015) Meng et al. (2016) [20,24]
Frog	Untargeted	HPLC-MS; NMR	Amino acid derivative (kynurenic acid)	Mariano et al. (2015) [26]
Frog	Untargeted	HPLC; NMR	Amino acids, sugars	Cavalante et al. (2017) [25]
Ant	Untargeted	GC-MS	Piperidine alkaloids	Lai et al. (2009) Chen et al. (2009) Chen et al. (2012) [34,35,36]
Wasp	Targeted	HPLC	Amino acids	[37]

HPLC high-performance liquid chromatography; HPLC-MS high-performance liquid chromatography coupled with mass spectrometry; DI-MS direct ionization mass spectrometry; UHPLC-MS ultra-high performance liquid chromatography coupled with mass spectrometry; NMR nuclear magnetic resonance; GC-MS gas chromatography-mass spectrometry.

**Table 2:** Bufadienolides identified in selected toad poisons [19,21,22].

Compound	*Bufobufo gargarizans*	*Bufo marinus*	*Bufo viridis*	*Rhinella crucifer*	*Rhinella marina*	*Rhinella major*	*Rhaebo guttatus*
Arenobufagin	+		*+*				
Bufotalin	+						
Bufalin	+	+	+	+	+		
Cinobufagin	+						
Cinobufotalin	+						
Dehydrobufotenine				+	+	+	+
Desacetylcinobufagin						+	+
Gamabufotalin	+		+				
Hellebrigenin	+		+	+		+	
Hellebrigenol-3-*O*-sulfate						+	
Marinobufagin	+	+	+	+	+	+	+
*N*′-*N*′-dimethyl-serotonin(Bufotenin)				+		+	
Resibufogenin	+	+					+
Telocinobufagin	+	+	+	+	+		
